# Zoonotic bacterial survey assessed by next-generation sequencing

**DOI:** 10.1186/1756-3305-7-S1-O14

**Published:** 2014-04-01

**Authors:** M Razzauti, M Galan, M Bernard, J Cheval, S Maman, N Charbonnel, M Vayssier-Taussat, M Eloit, J-F Cosson

**Affiliations:** 1 INRA, UMR CBGP (INRA ⁄ IRD ⁄ Cirad ⁄ Montpellier SupAgro), France; 2 INRA, GABI, Sigenae, Domaine de Vilvert, 78352 Jouy-en-Josas, France; 3 PathoQuest SAS, Paris, France; 4 INRA, LGC, Sigenae, Chemin de Borde rouge 31326 Castanet Tolosan, France; 5 INRA, UMR Bipar, Enva, Anses, USC INRA, Maisons-Alfort, France; 6 Ecole Nationale Vétérinaire d'Alfort, UMR 1161 Virologie ENVA, INRA, ANSES, Maisons-Alfort, France; 7 Institut Pasteur, Laboratory of Pathogen Discovery, Paris, France

## 

Rodents represent one of the major sources of pathogens; most of them are vectored by ticks. Tick-borne diseases are very diverse and cause a wide range of diseases in livestock and human populations. Rodents, carrying ticks, are distributed across a vast range of natural habitats and they often live in close contact with humans and their domestic animals, exposing them to zoonoses circulating in natural ecosystems. In this study, we analyse the potential of Next-Generation Sequencing (NGS) technologies as a tool for large-scale survey of bacterial zoonotic pathogens carried by rodents. We combined two NGS approaches in order to establish a list of zoonotic bacteria and to identify their distribution in individuals of rodents in natural populations.

Briefly, RNA/DNA were extracted from the spleen of 192 rodents collected in Northeast France. RNA from all samples was pooled and submitted to high throughput RNA sequencing (RNAseq). Succeeding *de novo *assembly, bacterial contigs were assigned to the closest already-known taxa, revealing a list of zoonotic bacteria for the whole sample. Parallel, DNA samples were submitted to meta-barcoding approach: each sample was amplified by PCR using universal primers tagged at the V4 region of the 16S rRNA. The amplified templates were multiplexed and submitted to 454-pyrosequencing. The resulting dataset was demultiplexed using a home-made pipeline that assigns each read to a sample using the tagged primers, following these were processed using Mothur pipeline to construct OTUs and classify them using the RDP database. These methods allowed listing bacteria detected in each rodent and, so derive the prevalence, coinfections and bacteria interactions.

DNA/RNA of the following bacteria genera were detected by both approaches, RNAseq and DNA 16S-metabarcoding: *Bartonella*, *Leptospira*, *Borrelia*, *Rickettsia, Treponema*, *Neisseria*, *Spiroplasma*, *Klebsiella, Listeria *and *Shigella*. Some unexpected genera were detected; such as *Orientia*, up to now only found in Asia or *Helicobacter*, generally thought to be restricted to animal guts. Several bacterial pathogens explored by RNAseq passed undetected by 16S-metabarcoding: *Anaplasma, (Neo)Ehrlichia, Wolbachia, Brucella, Coxiella, Campylobacter, Mycoplasma, Salmonella, Yersinia*, and *Francisella*. Furthermore, 16S meta-barcoding allowed to specify prevalence of bacteria within our sample, and revealed high level of coinfection in wild rodents.

Our data demonstrate that NGS allows having a rather complete screening of pathogenic bacteria present in animal reservoirs without any *a priori *on their presence, while having a price compatible with cohort studies. NGS approaches are becoming the new routine approaches in large-scale epidemiological studies.

